# Hypoglycaemic activity of ethanolic extract of *Garcinia mangostana* Linn. in normoglycaemic and streptozotocin-induced diabetic rats

**DOI:** 10.1186/s12906-016-1118-9

**Published:** 2016-05-21

**Authors:** Muhammad Taher, Tg Muhamad Faris Syafiq Tg Zakaria, Deny Susanti, Zainul Amiruddin Zakaria

**Affiliations:** Department of Pharmaceutical Technology, Faculty of Pharmacy, International Islamic University Malaysia, Jalan Sultan Ahmad Shah, 25200 Kuantan, Pahang Malaysia; Department of Chemistry, Faculty of Science, International Islamic University Malaysia, Jalan Sultan Ahmad Shah, 25200 Kuantan, Pahang Malaysia; Department of Biomedical Sciences, Faculty of Medicine and Health Sciences, Universiti Putra Malaysia, 43400 Serdang, Selangor Malaysia

**Keywords:** *Garcinia mangostana*, Hypoglycaemic effect, Streptozotocin, Single-dose, Glibenclamide

## Abstract

**Background:**

Various parts of *Garcinia mangostana* Linn., including its pericarp, have been traditionally used to treat a variety of ailments. In an attempt to establish its medicinal value, the present study was carried out to determine the hypoglycaemic potential of *G. mangostana* pericarp ethanolic extract (GME) using the streptozotocin-induced (STZ) diabetic rats.

**Methods:**

GME at 2,000 mg/kg was subjected to a single-dose acute toxicity test. Following this, the effect of GME (50, 100, and 200 mg/kg) on blood glucose level of normoglycaemic and STZ-induced diabetic rats was determined using single-dose (acute) and multiple-dose (subacute) approaches. Subsequent to the multiple-dose study, serum biochemical analysis and liver histopathological examination were also performed. Throughout the experiments, the effect of GME was compared against the standard hypoglycaemic drug, glibenclamide.

**Results:**

GME was safe for oral consumption up to the dose of 2,000 mg/kg. In both single- and multiple-dose studies, GME significantly (*p* < 0.05) reduced the blood glucose level in normoglycaemic rats and STZ-induced diabetic rats when compared against the normal control group or diabetic control group, respectively. Moreover, GME also significantly (*p* < 0.05) increased the rats’ body weight in comparison to the diabetic control group in the multiple-dose study. GME also significantly (*p* < 0.05) reduced the levels of certain biochemical parameters [i.e., triglycerides (TG), total cholesterol (TC), low density lipoprotein (LDL), very low density lipoprotein (VLDL), serum glutamic oxaloacetic transaminase (SGOT), serum glutamic pyruvic transaminase (SGPT), urea, and creatinine] while increased the others [i.e., high density lipoprotein (HDL) and total protein (TP)] when compared to the diabetic control group. Histopathological assessment of the collected liver revealed a mild increase in the population of β-cells in the diabetic rats.

**Conclusion:**

GME exerts the hypoglycaemic activity possibly by increasing the population of insulin-producing β-cells. This activity could be attributed to the presence of antioxidant-bearing tannins like epicathecin, and xanthones like α-mangostin. Thus, the findings demonstrated that GME could be a potential candidate in the management of diabetes owing to its hypoglycaemic effect.

## Background

The number of diabetic mellitus (DM) cases is rapidly increasing in recent years. According to the National Diabetes Statistics Report [1], approximately 29.1 million Americans, or 9.3 % of the population, had diabetes in 2012 with around 8.1 million of them were undiagnosed. Moreover, there were about 3.3 million cases of diabetes reported in Malaysia in 2015, according to the International Diabetes Federation [2]. Diabetes mellitus is a chronic disease characterised by the high blood glucose levels as a result of impaired glucose metabolism. Impaired glucose metabolism occurs when the pancreas does not produce enough insulin or when the body cannot effectively use the insulin it produces [3]. Hyperglycaemia, or raised blood sugar, is a general effect of uncontrolled diabetes that eventually leads to severe damage to many of the body’s systems, especially the nerves and blood vessels. Elevated blood glucose triggers oxidative damage of cell membranes through the production of superoxide anions, which generate hydroxyl radicals via Haber-Weiss reaction, inducing peroxidation of membrane lipids and protein glycation. These radicals further cause derangement of major biomolecules including carbohydrate, protein, lipid, and DNA [3]. Antioxidants play an important role in the prevention of cell damage against reactive oxygen species (ROS). Thus, herbal preparations containing both antioxidants and hypoglycaemic properties would be useful in diabetic management.

*Garcinia mangostana* Linn., generally known as mangosteen and belongs to the family Clusiaceae, is a tree cultivated for centuries in the tropical rainforest. Its fruit hull is used as phytomedicine in the Southeast Asia for the treatment of skin and wound infection [4], inflammation, diarrhoea [5], cholera, and dysentery [6]. Phytochemical studies of *G. mangostana* extracts (GME) report that this plant contains a variety of secondary metabolites such as oxygenated and prenylated xanthones [7]. Moreover, 50 xanthones have been identified in *G. mangostana* [8]. The ethnopharmacological views of the fruit rinds suggest remarkable properties such as antioxidants [9], antitumour [10], anti-inflammatory [11], analgesic [12], antiviral activities [13], cardioprotective effects [14], antifungal [15], antiallergy [16], antibacterial [17], antituberculosis [18], and immunomodulation [19]. Xanthone backbones of mangosteen have been reported as a potent molecular basis for *in vitro* α-glucosidase inhibition and proven to reduce postprandial hyperglycaemic condition by suppressing the glucose absorption [20]. The extract of *G. mangostana* has also been reported to retard glucose absorption through inhibition of carbohydrate-hydrolysing enzymes such as α-glucosidase and α-amylase [21]. The major component of the extract, α-mangostin, demonstrates a protective role in sexual dysfunction and exerts an antidiabetic effect in streptozotocin-induced diabetic male rats possibly via the reduction of blood glucose levels [22]. Preliminary studies in our laboratory indicated potent antioxidant and hypoglycaemic effects of crude ethanolic extracts on normoglycaemic models in rats. Hence, the present study was performed to evaluate the hypoglycaemic efficacy of crude ethanolic extracts of GME on the normal and streptozotocin-induced diabetic rats.

## Methods

### Plant identification

Taxonomic identification was made by Dr. Norazian Mohd Hassan, a botanist at the Faculty of Pharmacy, International Islamic University Malaysia (IIUM), Malaysia against the voucher specimen with the reference number of PIIUM-0201. The sample was also deposited in the Herbarium of the Kulliyyah of Pharmacy, IIUM, Malaysia.

### Drugs, chemicals, and reagents

The drugs used in the present study were glibenclamide and streptozotocin (STZ) (Sigma-Aldrich, St. Louis, MO, USA), while the chemicals used were ethanol, chloroform, diethyl ether, formaldehyde, and paraffin wax (Merck, Germany). All reagents used for serum biochemical analysis were procured from Thermo Fisher Scientific (VA, USA), while sodium carboxymethyl cellulose (Na CMC) and citrate buffer were obtained from R & M Chemicals (Canada). All chemicals and reagents used were of analytical grade.

### Plant material and extractions

Approximately 1.5 kg of mangosteen was obtained on June 2012 from the local wet market in Kuantan, Pahang, Malaysia. The pericarp was separated from its edible part and washed with clean sterile water to remove surface pollutants before being left to dry for 72 h at 37 °C in the laboratory drier (Memmert, Germany). The dried pericarp was then pulverised to powder form using an electric grinder and then further dried for another 24 h at room temperature. The powdered sample was then subjected to cold maceration in ethanol in the ratio of 4:1 (w:w) for 72 h at room temperature. The extracts were then filtered and evaporated to dryness at 50 °C under reduced pressure using the rotary evaporator (IKA, Germany) to yield a concentrated yellowish crude extract. The crude, dried ethanolic extract was subsequently stored at 2–8 °C until use.

### Experimental animals

Healthy adult male Sprague–Dawley rats (weighed 150–250 g; aged 16–19 weeks), obtained from the animal house of the Faculty of Medicine and Health Science, Universiti Putra Malaysia (UPM), Selangor, Malaysia, were used in the present study. Upon arrival, the animals were left to acclimatise for a week under laboratory conditions in the animal laboratory, Faculty of Pharmacy, IIUM, Malaysia. Initial body weights were recorded a day before the commencements of experiments. The rats were randomly assigned to 6 equal groups (*n* = 6) and housed separately in polypropylene cages. The animals were kept under standard environmental conditions (temperature 25 ± 2 °C, relative humidity 55 ± 10 %, and 12 h light/dark cycle) and were maintained under standard pellet diet and water *ad libitum*. All experiments were performed in accordance with the current ethical norms approved by the Institutional Animal Care and Use Committee (IACUC), Integrated Centre for Research Animal, Care & Use (ICRACU), IIUM, Malaysia (Approval no.: IIUM/IACUC Approval/2013/(1)(1)).

### Acute toxicity determination

The acute oral toxicity study was performed on healthy Sprague–Dawley rats of both sexes, according to the Organisation for Economic Co-operation and Development (OECD) Guideline no. 423. The rats were divided into two groups (*n* = 6). Group 1 served as the control group and received only vehicle, while Group 2 received fixed dose of 2,000 mg/kg of GME. The rats were fasted overnight before oral treatment of GME via force-feeding method. Following the extract administration, the animals were continuously observed for three hours for any changes in the autonomics and the general behavioural and neurological profiles. The observations were continued for the subsequent 24 h in three-hour intervals for mortality and daily for 14 days.

### Experimental design

Hypoglycaemic activity of GME was assessed in the normal and STZ-induced diabetic rats via single-dose study, while multiple-dose study was only performed on STZ-induced diabetic rats. The test samples were suspended in 0.2 % w/v Na CMC in distilled water. Glibenclamide (0.5 mg/kg) was used as a reference drug (control positive group) during the study. All the test samples were administered through oral route.

### Single-dose study (acute antihyperglycaemic model)

#### Evaluation of GME effect on normoglycaemic rats

The rats were randomly assigned into 5 groups of six animals each. Group 1 served as the normal control and received only vehicle, while Group 2 that served as the positive control group received 0.5 mg/kg glibenclamide. On the other hand, Group 3, 4, and 5 were orally administered with GME at the dose of 50, 100, and 200 mg/kg, respectively. All the rats were fasted for 16 h prior to treatment, but allowed a free access to water before and throughout the experiments. At the end of fasting period, taken as zero time (0 h), blood glucose level was measured by tail vein puncture at 1, 2, 4, and 8 h after the sample administration.

#### Evaluation of GME effect on STZ-induced diabetic rats

Rats weighing 150–250 g were selected and fasted for 16 h prior to experiments and allowed an access to water *ad libitum*. Experimental diabetes was induced by freshly dissolved STZ (50 mg/kg, i.p.) in cold citrate buffer (pH 4.5, 0.1 M). Diabetic rats were confirmed three days after the STZ injection. The rats with the fasting blood glucose of >250 mg/dL were considered diabetic and included in the study. The rats were randomly assigned into 5 groups of six animals each and then subjected to the following treatments: Group 1 served as diabetic control group and received only vehicle; Group 2 served as the positive control group and received 0.5 mg/kg glibenclamide; and Group 3, 4 and 5 received the test extract at the dose of 50, 100, and 200 mg/kg, respectively. All the test samples were administered in a similar manner. Blood glucose level was estimated at the interval of 1, 2, 4, and 8 h after the extract administration, respectively.

### Multiple-dose study (subacute antihyperglycaemic model)

#### Evaluation of GME on STZ-induced diabetic rats

Diabetes mellitus was induced by a single intraperitoneal injection of freshly prepared 50 mg/kg STZ dissolved in 0.1 M pH 4.5 cold citrate buffer in overnight-fasted rats (16 h). Blood glucose level was estimated prior to the injection of STZ and monitored for 24–72 h for confirmation of diabetes. Only rats with hyperglycaemia (blood glucose of >250 mg/dL) were included in the experiments. The blood concentration of glucose in normal rats should be within 80–110 mg/dL. The treatment commenced 4 days after the STZ injection, which was considered as Day 1 of treatment, and continued for another 28 days. The rats were divided into the following groups comprising six rats in each group:

Group 1: Normal control, received vehicle only.

Group 2: Diabetic control, received vehicle only.

Group 3: Diabetic rats, received 0.5 mg/kg glibenclamide (p.o.).

Group 4: Diabetic rats, administered with 50 mg/kg of GME1 orally.

Group 5: Diabetic rats, administered with 100 mg/kg of GME2 orally.

Group 6: Diabetic rats, administered with 200 mg/kg of GME3 orally.

### Blood collection

Overnight-fasted rats (16 h) were sacrificed by cervical decapitation on Day 29 of the experiments following the ethical norms granted by the ethics committee. The trunk blood was collected in serum separation tubes (BD Vacutainer® SST II), which contained clot activator. Blood clot was removed by centrifugation at 2,000 × g for 10 min in a refrigerated centrifuge CR4 22 (Jouan, France) at 4 °C. The resulting supernatant, designated as serum, was transferred into a clean polypropylene tube (Eppendorf, Germany) and maintained at 2–8 °C prior to the analysis.

### Biochemical analysis

The biochemical analysis was performed to measure the serum level of triglycerides (TG), total cholesterol (TC), high density lipoprotein (HDL), low density lipoprotein (LDL), very low density lipoprotein (VLDL), serum glutamic oxaloacetic transaminase (SGOT/AST), serum glutamic pyruvic transaminase (SGPT/ALT), alkaline phosphatase (ALP), urea, creatinine, and total protein (TP). Analytical tests were performed via UV–vis spectrophotometer using Konelab 20XTi (Thermo Fisher Scientific, USA).

### Histopathological assessment

The animals were sacrificed, and their pancreatic tissue, kidney, and liver were harvested and then fixed in 10 % (v/v) neutral buffered formalin. The fixed tissues or organs were then dehydrated and embedded in paraffin wax before being sectioned to approximately 5 μm thickness. Lastly, the sectioned tissues were stained with haematoxylin and eosin followed by the histopathological examinations.

### Statistical analysis

All values were expressed as mean ± S.E.M. The one-way analysis of variance (ANOVA) followed by the Tukey’s post hoc test was used to statistically analyse the data obtained when studying the single-dose effect of GME on the normoglycaemic or STZ-induced diabetic rats. On the other hand, the two-way ANOVA followed by the Bonferroni’s post hoc test was used to statistically analyse the data obtained when studying the multiple-dose effect of GME on blood glucose and body weight in the STZ-induced diabetic rats. The results were considered statistically significant at *p* < 0.05. The statistical package of IBM SPSS Statistics 21 for Windows was used in the analysis.

## Results

### Acute toxicity study

GME-treated animals of both sexes did not show any alterations in their neurobehavioural patterns, health status, or mortality at the fixed dose of 2,000 mg/kg. When compared to vehicle-treated group, there were no significant changes in the body weight and food intake. Hence, the extract was suggested to be safe for consumption even at the dosage of 2,000 mg/kg.

### Effect of GME on normoglycaemic rats (single-dose study)

**Fig. 1:**
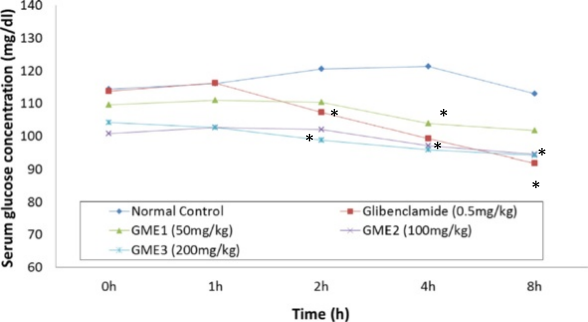
Effect of GME on normoglycaemic rats. Data are expressed as mean ± S.E.M. for six separate observations (*n* = 6); One-way ANOVA test followed by Tukey’s post-hoc test was used to analyse the data; *significantly different (*p* < 0.05) when compared to the normal control

Figure 1 shows the effect of orally administered GME at various doses on the blood glucose level in the normal healthy rats. The blood glucose level of normal control group was insignificantly maintained between 110 and 120 mg/dL, and pretreatment with 0.5 mg/kg glibenclamide significantly (*p* < 0.05) reduced the blood glucose level starting 2 h after the drug administration. Pretreatment with all doses of GME significantly (*p* < 0.05) reduced the blood glucose level starting 2 h (50 mg/kg GME) or 1 h (100 and 200 mg/kg GME) after the extract administration. The decrease in blood glucose level following the glibenclamide or GME administration was found to continue until the end of experiment. The blood glucose was expected to restore to normal after 8 h.

### Effect of GME on STZ-induced diabetic rats (single-dose study)

**Fig. 2:**
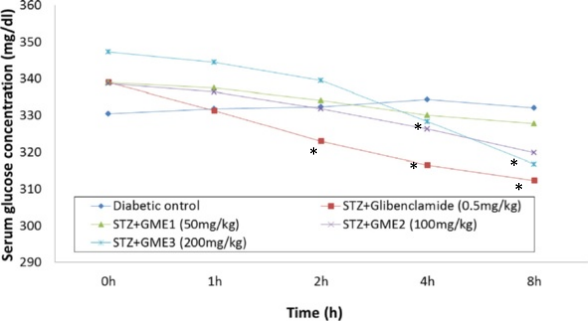
Effect of GME on STZ-induced diabetic rats. Data are expressed as mean ± S.E.M. for six separate observations (*n* = 6); One-way ANOVA test followed by Tukey’s post-hoc test was used to analyse the data; *significantly different (*p* < 0.05) when compared to the diabetic control

Figure 2 presents the effect of orally administered GME at various doses on the blood glucose levels in the STZ-induced diabetic rats. Treatment with 0.5 mg/kg glibenclamide caused a significant (*p* < 0.05) decrease in the blood glucose levels after 2 h of its administration. In comparison to the diabetic control group (untreated), the level continued to be lower until the end of experiments (8 h). Treatment with GME was also able to significantly (*p* < 0.05) reduce the blood glucose level at 200 mg/kg starting at the fourth hour after the extract administration.

### Effect of GME on blood glucose level and body weight changes in STZ-induced diabetic rats (multiple-dose study)

**Fig. 3:**
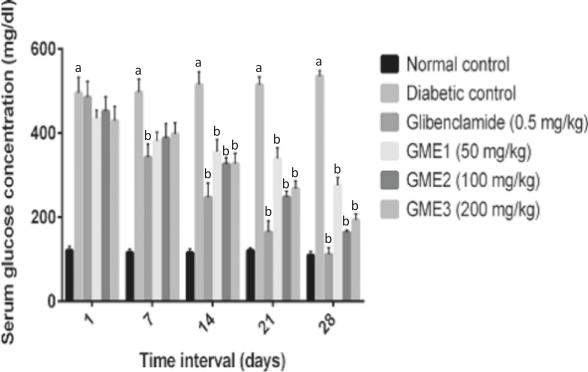
Effect of GME on blood glucose level in STZ-induced diabetics rats for multiple-dose study. Each value is expressed as mean ± S.E.M. (*n* = 6). Data analysis was performed by two-way ANOVA followed by Bonferroni’s post-hoc test. ^a^significantly different (*p* < 0.05) when compared to the normal control group; ^b^significantly different (*p* < 0.05) when compared to the corresponding values of the diabetic control group

**Fig. 4:**
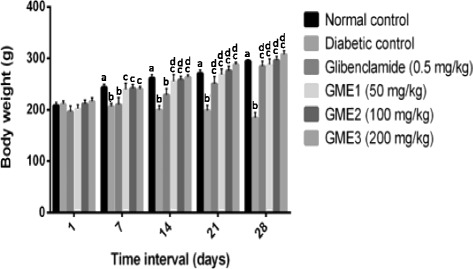
Effect of body weight in STZ-induced diabetic rats for 28 days of treatment. Each value is expressed as mean ± S.E.M. (*n* = 6). Data analysis was performed by two-way ANOVA followed by Bonferroni’s post-hoc test. ^a^significantly different (*p* < 0.05) when compared to the normal control group Day 1; ^b^significantly different (*p* < 0.05) when compared to the normal control group of the respective tie interval; ^c^significantly different (*p* < 0.05) when compared to the group treated with the respective test solution on Day 1; ^d^significantly different (*p* < 0.05) when compared to the diabetic control of the respective time interval

Figures 3 and 4 respectively depict the subacute effect of GME at various doses on the blood glucose level and body weight changes in STZ-induced diabetic rats. The diabetic control group demonstrated a significant (*p* < 0.05) increase in the blood glucose level starting from Day 1 while showing a significant (*p* < 0.05) decrease in their body weight from Day 14 when compared to the normal control group. Treatment with 0.5 mg/kg glibenclamide caused a significant (*p* < 0.05) and gradual decrease in the blood glucose level starting from Day 7 while significantly (*p* < 0.05) increasing the body weight of rats starting from Day 14 when compared to the diabetic control group. The significant effect of glibenclamide on both parameters continued to be seen until the end of experiment (Day 28). In the groups treated with GME, at all doses, a significant (*p* < 0.05) reduction of blood glucose level, but an increase in body weight changes, was observed on Day 14 until the end of experiment when compared to the diabetic control group. The decrease in glucose level by GME occurred in a dose-independent manner.

### Effect of GME on serum biochemical indices

#### Effect of GME on serum lipid profiles in STZ-induced diabetic rats

Table 1 presents the effect of GME at various doses on the serum lipid profiles in STZ-induced diabetic rats. There was a significant (*p* < 0.05) increase in the level of TG, TC, LDL, and VLDL in the diabetic control rats when compared to the normal control group. Pretreatment with GME or glibenclamide at all doses was found to significantly (*p* < 0.05) reduce the level of TG, TC, LDL, and VLDL in the STZ-induced diabetic rats.

**Table 1:** Effect of GME at various doses on the serum lipid profiles in STZ-treated diabetic rats

Experimental group	TG (mg/dL)	TC (mg/dL)	HDL (mg/dL)	LDL (mg/dL)	VLDL (mg/dL)
Normal control	85.00 ± 3.20	73.83 ± 4.35	15.50 ± 0.57	41.34 ± 4.45	17.00 ± 0.64
Diabetic control	219.5 ± 7.66^*^	133.5 ± 3.15^*^	11.46 ± 1.31	78.14 ± 4.61^*^	43.9 ± 1.53^*^
STZ + 0.5 mg/kg glibenclamide	104.17 ± 3.48^**^	71 ± 3.53^**^	20.26 ± 1.23^**^	29.91 ± 4.00^**^	20.83 ± 0.70^**^
STZ + 50 mg/kg GME	159.67 ± 5.23^**^	92 ± 2.32^**^	13.42 ± 1.28	46.65 ± 3.47^**^	31.93 ± 1.05^**^
STZ + 100 mg/kg GME	131.67 ± 5.37^**^	91.5 ± 2.17^**^	12.92 ± 1.02	52.25 ± 2.20^**^	26.33 ± 1.07^**^
STZ + 200 mg/kg GME	133.17 ± 3.40^**^	73.67 ± 4.06^**^	15.5 ± 1.20	31.53 ± 4.34^**^	26.63 ± 0.68^**^

^*^significantly different (*p* < 0.05) when compared to the normal control group of the respective column

^**^ significantly different (*p* < 0.05) when compared to the diabetic control group of the respective column

Data are expressed as mean ± S.E.M. for six separate observations (*n* = 6); One-way ANOVA test followed by Tukey’s post-hoc test (*p* < 0.05) was used to analyse differences among the selected lipid profiles

#### Effect of GME on kidney, total protein, and liver enzymes in STZ-induced diabetic rats

Table 2 shows the effect of GME at various doses on the liver and kidney biochemical parameters in the STZ-treated diabetic rats. With regard to the liver, the levels of SGOT, SGPT, and ALP significantly (*p* < 0.05) increased in the diabetic control rats when compared to the normal control rats. Pretreatment of STZ-induced diabetic rats with GME significantly (*p* < 0.05) reduced the level of SGOT and SGPT, but not ALP. With regard to the kidney, the diabetic control group showed a significant (*p* < 0.05) increase in the level of urea and creatinine, but there was a significant (*p* < 0.05) decrease in the level of TP. All the biochemical parameters of the kidney were significantly (*p* < 0.05) reversed after the treatment with GME or glibenclamide.

**Table 2:** Effect of GME on kidney profiles, SGOT, SGPT, ALP, and total protein in STZ-treated diabetic rats

Experimental group	SGOT (U/L)	SGPT (U/L)	ALP (U/L)	Urea (mg/dL)	Creatinine (mg/dL)	TP (g/dL)
Normal control	22.5 ± 2.46	64.5 ± 6.25	74.78 ± 5.40	22.5 ± 2.07	0.81 ± 0.03	7.12 ± 0.15
Diabetic control	76 ± 5.73^*^	159.5 ± 7.08^*^	173.5 ± 15.51^*^	143.7 ± 7.57^*^	1.25 ± 0.13^*^	5.05 ± 0.24^*^
STZ + 0.5 mg/kg glibenclamide	38.17 ± 3.62^**^	66.83 ± 5.46^**^	147.83 ± 15.90	54.25 ± 3.46^**^	0.99 ± 0.04	7.85 ± 0.24^**^
STZ + 50 mg/kg GME	87.5 ± 12.46	137.83 ± 6.28	156.67 ± 13.19	135.3 ± 3.86	0.84 ± 0.07^**^	6.15 ± 0.26^**^
STZ + 100 mg/kg GME	52 ± 3.91^**^	125.33 ± 6.86^**^	149.5 ± 11.84	96.6 ± 2.28^**^	0.98 ± 0.07	6.75 ± 0.13^**^
STZ + 200 mg/kg GME	56.17 ± 5.43^**^	148.5 ± 12.16	157.83 ± 14.91	105.77 ± 4.05^**^	0.87 ± 0.041^**^	6.72 ± 0.14^**^

^*^significantly different (*p* < 0.05) when compared to the normal control group of the respective column

^**^ significantly different (*p* < 0.05) when compared to the diabetic control group of the respective column

Data are expressed as mean ± S.E.M. for six separate observations (*n* = 6); One-way ANOVA test followed by Tukey’s post-hoc test (*p* < 0.05) was used to analyse differences among the selected kidney’s profiles

### Histopathological studies

**Fig. 5:**
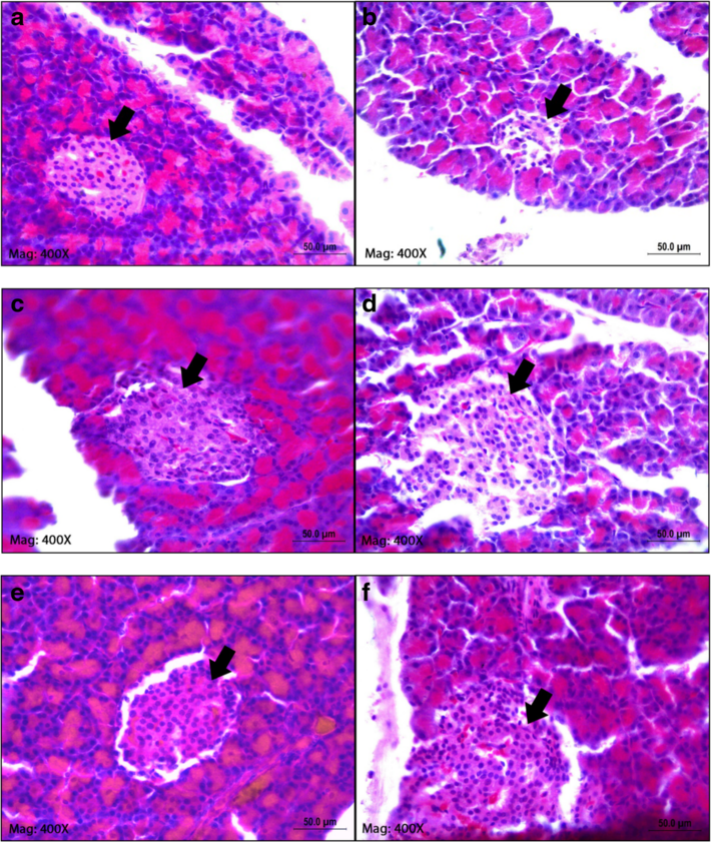
Photomicrograph of pancreatic islets of experimental rats after 28 days of treatment. **a** Normal control: Depicted typical rat pancreas with blood capillaries surrounded by centroacinar cells which contain serous acini. **b** Diabetic control: Observable necrosis and degranulation of serous acini resulted in diminished number of β-cells and islets size. **c** STZ-glibenclamide: Granulated islets, hyperplasia and restoration of normal cytoarchitecture with regeneration of pancreatic islets. **d** STZ-GME1: Exocrine region and islets of Langerhans, with scattered β-cells and visible red blood cells. **e** STZ-GME2: Hyperplasia, granulated islets and mild expansion of pancreatic islets. **f** STZ-GME3: Evenly distributed and increased number of β-cells with moderate rejuvenated pancreatic islet. (All samples were stained with haematoxylin and eosin, original magnification × 400)

At the end of the 28-day treatment, histopathological assessment was carried out on the rats’ pancreas (see Fig. 5). The microscopic analysis of liver tissue obtained from the normal control group showed the presence of a normal pancreatic tissue consisting of the normal acini, normal cellular population, and absence of damaged islets or hyperplasia. Tissue from the diabetic control group demonstrated the presence of partial islets destruction with reduced size and observable flattening of nuclei, which were pushed to the periphery. The tissue obtained from glibenclamide-treated group revealed an obvious improvement with the recovery of normal pancreatic histoarchitecture. As for the group treated with GME, the tissue treated with 50 mg/kg GME showed the restoration of normal cellular population size of islets of Langerhans. The treatment also reduced the damaged islets and decreased the presence of hyperplasia. As for the group treated with 100 mg/kg GME, the tissue showed the presence of granulated islets of Langerhans with prominent hyperplasia. In the 200 mg/kg GME-treated group, the tissue demonstrated the presence of evenly distributed β-cells in an increase number and the absence of damaged islets and hyperplasia.

## Discussion

Diabetes mellitus is a metabolic disorder characterised by derangement in carbohydrate, protein, and fat metabolisms resulting from impairment in glucose homeostasis, lack of insulin secretion, and development of macro- and microvascular dysfunctions [23]. Modern antidiabetic agents like sulphonylureas, thiazolidinediones, and biguanides are commercially prescribed but failed to provide a prolonged glycaemic control effect. Medicinal plants have been widely used to treat diabetes due to their effectiveness, safety, affordability, and acceptability. The present study highlighted the potential hypoglycaemic action of GME in normal and diabetic-induced rats. In this study, diabetes was induced in the rats using 50 mg/kg STZ, a glucosamine derivative of nitrosurea, which selectively destroys pancreatic islets of β-cells and causes development of hyperglycaemia and glycosuria, as seen in type 1 diabetic patients. Glibenclamide (glyburide), a member of the second generation sulphonylureas, provides an effective treatment for patients with moderate diabetes. Other than its glucose-lowering properties, glibenclamide seems to have antioxidant properties and is capable of restoring liver antioxidant enzymes, superoxide dismutase (SOD), and catalase (CAT) in STZ-induced diabetic rats [24]. The increase in blood glucose level might be due to shortage of insulin as a result of destruction of pancreas interceded by STZ action, which boosts ATP dephosphorylation, which in turn generates superoxide anions, hydrogen peroxide, and hydroxyl radicals. Elevated intracellular peroxides in pancreatic islets further mediate a damage induced by reactive oxygen species (ROS) [25]. Under hyperglycaemic conditions, antioxidants are supposed to regenerate damaged extracellular matrix proteins and cell growth as a result of ROS elevation through nonenzymatic glycation of proteins, as well as through auto-oxidation [26]. This indication marks the increased levels of indicators of oxidative stress in diabetic patients. Because oxidative stress is associated with pathogenesis of diabetes, therefore, it can be postulated that the antioxidants may exert a key role in the management of DM.

Mangosteen’s pericarps, leaves, and barks have been used traditionally to treat various ailments such as arthritis, diarrhoea, dysentery, inflammation, and skin disorders, besides being applied in the healing of wound [27]. However, no study has addressed its hypoglycaemic effect although it has been commercially claimed as an antidiabetic agent. To date, numerous articles have reported the use of *in vitro* assays to evaluate the pharmacological effect of *G. mangostana*’s purified compounds mainly α-mangostin, which is the major bioactive secondary metabolite of xanthone derivatives [28] and the first substance to be isolated [29]. There are limited animal models testing on α-mangostin, which could be attributed to its low bioavailability via oral treatment when compared to the i.v. administration [28]. Although there is an issue of low bioavailability when α-mangostin is given orally, GME is still given orally and tested in diabetic animal models as its beneficial bioactive components consist of not only pure conjugated xanthones, but also a combination of free and unconjugated compounds [30]. This combination, as far as the pharmacokinetic is concerned, can improve the bioavailability of GME. This fact could be one of the best evidences of better health-promoting properties of the extract over the administration of pure compounds like xanthones. Furthermore, the extract has been reported to exhibit α-glucosidase inhibitory activity, which has been proven to reduce postprandial hyperglycaemia by inhibiting glucose absorption [31]. Moreover, the ethanol extract of *G. mangostana* also shows a significant hypoglycaemic effect following the oral maltose administration [20], and the effect is similar to the reference drug, acarbose [32].

In this study, for the normoglycaemic rats, significant blood glucose-lowering actions were observed in GME- and glibenclamide-treated groups when compared to the normal control group. The effect of GME on normoglycaemic animals suggests that the pericarp of *G. mangostana* has a mild lowering effect on normal glucose levels. This effect was comparable to that of glibenclamide, an insulin secretagogue, which also lowers blood glucose in normal animals. Provided the β-cells are fully functional, sulphonylureas such as glibenclamide can cause hypoglycaemia because insulin release is initiated even when glucose concentrations are below the normal threshold for glucose-stimulated insulin release (approximately 5 mmol/L or 90 mg/dL) [33]. In the same study on STZ-induced rats, we reported the increase of hypoglycaemic action of glibenclamide and GME in a dose-dependent manner. This was indicative of the potentiation of GME on insulin secretion and the enhanced peripheral utilisation of insulin in diabetic rats as supported by the histopathological findings. Glibenclamide was used as a reference hypoglycaemic agent to compare the efficacy of a variety of glucose-reducing compounds via enhanced activity of β-cells of the pancreas resulting in the secretion of larger amounts of insulin. In the multiple-dosage study, the elevated blood glucose improved after 28 days of GME treatment, suggesting that the extract’s antioxidant activity mediated the protective effect of β-cells in the diabetic rats. It is widely accepted that the enhancement of antioxidant activity could be one of the mechanisms responsible in the prevention of diabetic complications [34].

The STZ-induced rats initially showed a reduction in their body weight, suggesting abnormal water and food intake. However, the administration of GME in diabetic rats improved their weight commencing from Day 14 onwards in comparison to the diabetic control group. The increase in body weight could be due to amelioration of glycaemic control and structural proteins synthesis [35]. This finding was actually contradicting the finding made by Chivapat et al. [36], who reported that GME at the dose of 1,000 mg/kg or above caused reduction in the body weight of male rats. This observation was related to the effect of condensed tannins in the extract of *G. mangostana* pericarp [37] that are believed to be present in a high quantity due to the high dose (1,000 mg/kg) of GME used [36]. In other studies, the mice fed with a diet containing tannic acid underwent a growth retardation [38], while those fed with a high dose of tannins suffered from a slower growth rate when compared to the mice that consumed a low dose of tannins [39]. It is plausible to suggest that the amount of condensed tannins present in the GME at the dose of 50–200 mg/kg as used in the present study was not enough to trigger body weight reduction. In addition, weight loss in the diabetic rats could be the result of high catabolic rate of protein to amino acid for gluconeogenesis during insulin deficiency, which could be enhanced in the presence of enough concentration of tannins [39]. In the present study, the lack of protein catabolism could be related to the insufficient amount of condensed tannins present in the GME.

The high cholesterol and lipid levels mark an increased risk of atherosclerosis and coronary heart diseases development, which are considered secondary complications of diabetes [40, 41]. In the present study, GME was found to reduce the total cholesterol and triglycerides in STZ-treated rats, suggesting the extract’s capability to reduce atherogenic-related complications. Liver plays a crucial function in the metabolism, storage, detoxification, and excretion of xenobiotics and their metabolites. Liver function tests provide vital indicators of liver activities, which include SGOT, SGPT, and ALP [42]. High levels of these enzymes in the blood indicated leakage from the liver cytosol into the bloodstream, indicating a liver injury [42]. In the present study, GME reduced the levels of important liver enzymes, particularly SGOT and SGPT, in all the treated groups. Because of the promising reduction of liver transaminases, long-term studies should be conducted to observe the improvement of liver function markers following the consumption of GME. It is important to highlight that the effect of GME on the level of liver enzymes was observed in a dose-independent manner with the maximum reduction observed at the dose of 100 mg/kg. This observation could be associated with the phenomenon known as “therapeutic windows” [43], which could possibly be related to the optimal hepatoprotective effect of GME or reduction in the enzyme activity. Other than that, GME was also observed to cause an increase in the urea and creatinine levels, which were reduced in the diabetic groups. The ability of diabetic rats to decrease both parameters seems to suggest that diabetes could lead to renal dysfunction. The significant improvement in the level of renal markers indicated the potential of GME to ameliorate the kidney dysfunction. Additionally, the total protein content was also found to improve in the diabetic rats following a treatment with GME, suggesting the remedial role of the extract towards the kidney function in the diabetic rats. Histopathological examination revealed an improved preservation of normal pancreatic islets and diminished necrotic changes as compared to the STZ-induced diabetic rats. The effect of GME might be due to the pancreatic rejuvenation via enhanced protein synthesis, accelerated detoxification, potentiation of antioxidant defence, and neutralisation of free radicals, which confirmed the insulinotropic effect of GME through the regeneration of insulin-producing β-cells.

The hypoglycaemic activity of GME could also be attributable to the extract’s phytoconstituents. Polyphenolics like tannins have been reported to be present in the pericarp of *G. mangostana* [44]*.* In addition, several polyphenols have been identified from the pericarp namely 4-aryl-2-flavanylbenzopyran derivative, 3,4,3′,5’-tetrahydroxy-5-methoxybenzophenone, 2,3-dihydrochromone derivative, epicatechin, and procyanidin B2 [45]. Previous studies have also reported the presence of various types of xanthones in different parts of *G. mangostana*, including the pericarp. Some of the identified xanthones derived from pericarp are mangostinone, α-mangostin, β-mangostin, γ-mangostin, gartanin, garcinone E, 1,5-dihydroxy-2-(3-methylbut-2-enyl)-3- methoxyxanthone, 1,7-dihydroxy-2-(3-methylbut-2-enyl)-3-methoxyxanthone [46], 8-hydroxycudraxanthone G, mangostingone, cudraxanthone G, 8-deoxygartanin, garcimangosone B, garcinone D, garcinone E, gartanin, 1-isomangostin, mangostinone, smeathxanthone A, tovophyllin A, mangostanaxanthones I, mangostanaxanthones II, 9-hydroxycalabaxanthone, parvifolixanthone C, rubraxanthone [47], 1,3,7-trihydroxy-2-(3-methyl-2-butenyl)-8-(3-hydroxy-3-methylbutyl)-xanthone, 1,3,8-trihydroxy-2-(3-methyl-2-butenyl)-4-(3-hydroxy-3-methylbutanoyl)-xanthone, garcinones C, garcinones D, gartanin, xanthone I [48], garcimangosxanthone F (1), garcimangosxanthone G [49], and mangostanate [50].

The presence of xanthones and polyphenols like tannins in the pericarp of *G. mangostana* could also be used to explain the hypoglycaemic activity of GME observed. Tannins, one of the major groups of antioxidant polyphenols, can be classified into two broad groups namely hydrolysable tannins and condensed tannins. Hydrolysable tannins are molecules with a polyol (D-glucose) as a central core. The hydroxyl groups of these carbohydrates are partially or totally esterified with phenolic groups (i.e., gallic acid or ellagic acid). Hydrolysable tannins are usually present in low amounts in plants and are easily hydrolysed by mild acids and bases to yield carbohydrate and phenolic acids. Condensed tannins are a group of naturally occurring polyphenolic bioflavonoids, specifically taking the form of oligomers or polymers of polyhydroxy flavan-3-ol units such as (+)-catechin and (−)-epicatechin. Condensed tannins are more common in the plant kingdom and have been reported to have a wide range of biological and pharmacological activities including antioxidative activity without inducing significant toxicological effects [51]. These protective effects are related to their capacity (a) to act as free radical scavengers and (b) to stimulate antioxidant enzymes. Moreover, tannins have been observed to improve the glucose uptake through mediators of the insulin-signalling pathways (i.e., phosphoinositide 3-kinase (PI3K) and mitogen-activated protein kinase (p38 MAPK) activations and GLUT-4 translocation. The reduction in glycaemia (blood glucose levels) caused by tannins and other phenolic compounds has been attributed to actions such as (a) a decrease in the absorption of nutrients (b) a decrease in food intake (c) initiation of β-cell regeneration, (d) direct action on adipose cells that enhances insulin activity, and (e) sharing insulin-signalling pathways in hepatocytes possibly by modulation of the redox state of cell [51]. In other words, tannins work to establish an organised network of metabolic sensors that incorporates glucose homeostasis, lipid metabolism, inflammation, drug metabolism, bile acid synthesis, and some other processes. One of the restorative approaches to decrease postprandial hyperglycaemia is by preventing or delaying the absorption of glucose via inhibition of carbohydrate hydrolysing enzymes, α-amylase, and α-glucosidase in the digestive organs. Interestingly, tannins are natural inhibitors of α-amylase and α-glucosidase with a potent inhibitory effect on the latter, but a mild inhibitory effect on the former. Therefore, phenolic antioxidant-mediated inhibition of these enzymes can significantly decrease the postprandial hyperglycaemia after ingestion of a mixed carbohydrate diet and could be an effective strategy in the control of diabetes [51]. Epicatechin, one of the natural condensed tannins present in *G. mangostana* pericarp [45], prevents hyperglycaemia by inducing β-cell regeneration [52]. This effect of epicatechin is supported by the histopathological examination of the rat’s liver tissue treated with GME in comparison to the tissue of diabetic rats. Taking other reports into consideration, several other possible mechanisms of hypoglycaemic condition could also be suggested to be triggered by tannins in order to induce hypoglycaemia, and these mechanisms include (a) reduction in food intake, (b) inhibition of intestinal glucose absorption, and (c) direct action on adipose cells by enhancing insulin activity in rat epididymal adipocytes. Catechins are powerful antioxidants that inhibit oxidation of LDL-cholesterol, reduce cholesterol levels, and reduce body fat, resulting in a decreased risk of heart disease.

Other than that, several xanthones have also been reported to exert antihyperglycaemic activity. According to Jariyapongskul et al. [53], the damage in the retinal microvasculature in type 2 diabetic rat model such as reduction of ocular blood flow (OBF) and leakage of the blood-retinal barrier (BRB) permeability is associated with hyperglycaemia and the accumulations of free radicals, advanced glycation end products (AGEs), receptor of advanced glycation end products (RAGE), tumour necrosis factor alpha (TNF-*α*), and vascular endothelial growth factor (VEGF) levels in the retinal tissues. α-Mangostin has also been reported to prevent retinal microvascular complications in diabetic rats possibly via its antihyperglycaemic, antioxidant, anti-inflammatory, and antiglycation properties [53]. Other xanthone, mangiferin reduces the level of blood glucose by increasing the level of insulin in STZ-induce diabetic rats. Moreover, mangiferin also increases the level of hexokinase, pyruvate kinase, glucose-6-phosphate dehydrogenase, glycogen synthase, and glycogen content to near normal in diabetic rats. The levels of these enzymes are reduced following a treatment with STZ. On the other hand, the activities of lactate dehydrogenase, glucose-6-phosphatase, fructose-1,6-diphosphatase, and glycogen phosphorylase in the liver tissue of diabetic rats are decreased after a treatment with mangiferin [54]. Besides, mangiferin has also been shown to exert an antidiabetic activity in KK-Ay mice (an animal model of type-2 diabetes) by decreasing insulin resistance [55]. Moreover, several xanthones isolated from the fruit case of *G. mangostana* have also been reported to exert α-glucosidase inhibitory activity [56].

## Conclusion

The ethanolic extract of *G. mangostana* pericarp exerts hypoglycaemic and antidiabetic activities possibly by increasing the population of insulin-producing β-cells. This observation could further be attributed to the presence of antioxidant-bearing tannins like epicathecin and xanthones like α-mangostin that might act synergistically to exert the effect. Thus, the finding demonstrated that GME could be a potential candidate in the management of diabetes owing to its hypoglycaemic and thus antidiabetic effects. Therefore, further studies are required to elucidate the mechanism of action involved.

### Ethics approval and consent to participate

All experiments were performed in accordance with the current ethical norms approved by the Institutional Animal Care and Use Committee (IACUC), Integrated Centre for Research Animal, Care & Use (ICRACU), IIUM, Malaysia (Approval no.: IIUM/IACUC Approval/2013/(1)(1)).

### Consent for publication

Not applicable.

### Availability of data and materials

The supporting materials can be obtained upon request via email to the corresponding author.

## Abbreviations

AGE: advanced glycation end products
ALP: alanine aminotransferase
ATP: adenosine triphosphate
BRB: blood-retinal barrier
CAT: catalase
DM: diabetic mellitus
GLUT-4: glucose transporter-4
GME: *G. mangostana* pericarp ethanolic extract
HDL: high density lipoprotein
i.v: intravenous
LDL: low density lipoprotein
MAPK: mitogen-activated protein kinase
Na CMC: sodium carboxymethyl cellulose
OBF: ocular blood flow
PI3K: phosphoinositide 3-kinase
RAGE: receptor of advanced glycation end products
ROS: reactive oxygen species
SGOT: serum glutamic oxaloacetic transaminase
SGPT: serum glutamic pyruvic transaminase
SOD: superoxide dismutase
STZ: streptozotocin
TC: total cholesterol
TG: triglycerides
TNF-α: tumour necrosis factor alpha
TP: total protein
VEGF: vascular endothelial growth factor
VLDL: very low density lipoprotein
